# Exploring the antioxidant activity of Fe(III), Mn(III)Mn(II), and Cu(II) compounds in *Saccharomyces cerevisiae* and *Galleria mellonella* models of study

**DOI:** 10.1093/femsyr/foad052

**Published:** 2023-12-20

**Authors:** Larissa M M Mattos, Hyan M Hottum, Daniele C Pires, Bruna B Segat, Adolfo Horn, Christiane Fernandes, Marcos D Pereira

**Affiliations:** Departamento de Bioquímica, Instituto de Química, Universidade Federal do Rio de Janeiro, RJ, Brazil; Rede de Micologia RJ - FAPERJ; Departamento de Bioquímica, Instituto de Química, Universidade Federal do Rio de Janeiro, RJ, Brazil; Rede de Micologia RJ - FAPERJ; Departamento de Bioquímica, Instituto de Química, Universidade Federal do Rio de Janeiro, RJ, Brazil; Rede de Micologia RJ - FAPERJ; Departamento de Química, Universidade Federal de Santa Catarina, Florianópolis, SC, Brazil; Departamento de Química, Universidade Federal de Santa Catarina, Florianópolis, SC, Brazil; Departamento de Química, Universidade Federal de Santa Catarina, Florianópolis, SC, Brazil; Departamento de Bioquímica, Instituto de Química, Universidade Federal do Rio de Janeiro, RJ, Brazil; Rede de Micologia RJ - FAPERJ

**Keywords:** oxidative stress, coordination compounds, antioxidants, *Saccharomyces cerevisiae*, *Galleria mellonella*

## Abstract

Reactive oxygen species (ROS) are closely related to oxidative stress, aging, and the onset of human diseases. To mitigate ROS-induced damages, extensive research has focused on examining the antioxidative attributes of various synthetic/natural substances. Coordination compounds serving as synthetic antioxidants have emerged as a promising approach to attenuate ROS toxicity. Herein, we investigated the antioxidant potential of a series of Fe(III) (1), Mn(III)Mn(II) (2) and Cu(II) (3) coordination compounds synthesized with the ligand N-(2-hydroxybenzyl)-N-(2-pyridylmethyl)[(3-chloro)(2-hydroxy)]-propylamine in *Saccharomyces cerevisiae* exposed to oxidative stress. We also assessed the antioxidant potential of these complexes in the alternative model of study, *Galleria mellonella*. DPPH analysis indicated that these complexes presented moderate antioxidant activity. However, treating *Saccharomyces cerevisiae* with 1, 2 and 3 increased the tolerance against oxidative stress and extended yeast lifespan. The treatment of yeast cells with these complexes decreased lipid peroxidation and catalase activity in stressed cells, whilst no change in SOD activity was observed. Moreover, these complexes induced the Hsp104 expression. In *G. mellonella*, complex administration extended larval survival under H_2_O_2_ stress and did not affect the insect's life cycle. Our results suggest that the antioxidant potential exhibited by these complexes could be further explored to mitigate various oxidative stress-related disorders.

## Teaser

The antioxidant activity of coordination compounds derivatives of the ligand H2BPClNOL was evaluated. Complexes increased *Saccharomyces cerevisiae* and *Galleria mellonella* tolerance against oxidative stress. Complexes extended yeast lifespan, reduced lipid peroxidation, and CAT activity under H_2_O_2_ stress, and promoted the induction of Hsp104 expression.

## Introduction

Oxidative stress is characterized by the excessive production of oxidizing agents, such as reactive oxygen (ROS) and nitrogen (RNS) species, which are counteracted by the presence of antioxidants. The disruption of redox balance is multifactorial, and it has been related to endogenous (e.g. mitochondrial function and the activity of xanthine, cytochrome P450, monoamine and NADPH oxidases) and exogenous (e.g. exposure to tobacco, medications, UV and/or ionizing radiation) factors (Sies 2015; Poprac *et al.*^2017^, During oxidative stress, ROS-induced reactions may cause dramatic changes in various biological structures that are currently associated with the incidence of various oxidative stress-related diseases, such as cancer and neurodegenerative diseases (e.g. Alzheimer´s and Parkinson´s disease) (Valko et al. 2007, Sies 2015, Poprac et al. 2017; Tan *et al*. 2018. In this scenario, it is imperative to develop new strategies to mitigate ROS reactivity and the oxidative damages in proteins, lipids and nucleic acids.

All aerobic organisms have evolved a sophisticated antioxidant system, composed of enzymatic components [e.g. superoxide dismutases (SODs), catalases (CATs), glutathione peroxidases (GPXs) and peroxiredoxins (PRXs), methionine sulfoxide reductase (MsrA), 8-Oxoguanine DNA Glycosylase (OGG1) and mutY DNA glycosylase (MUTYH)], as wells as non-enzymatic biomolecules [e.g. glutathione (GSH), vitamins C, E and A, and thioredoxin (TRx)]. These elements play a crucial role in ROS elimination or redox signaling (Benzie 2000, Valko et al. 2007, Birben et al. 2012). The perfect functioning of this antioxidant system is critical for an effective cellular response against ROS. Among the well-known enzymatic antioxidant representatives, GPXs, CATs and SODs metalloenzymes are considered the primary antioxidant barrier protecting cells from the high reactivity of ROS (Greenwald 1990, Birben et al. 2012, Ighodaro and Akinloye 2018, Sepasi Tehrani and Moosavi-Movahedi 2018). It should be noted that these enzymes share a common feature; they all have a metal center in their active site. In the GPx enzyme, selenium is represented by the selenium derivative of cysteine, selenocysteine (Sec), while CATs have the protoporphyrin IX group containing iron(III) or a dinuclear active site of manganese. SODs may present different metals (e.g. iron, manganese, copper or nickel) at their active sites (Sarma and Mugesh 2008, Batinic-Haberle et al. 2014, Sepasi Tehrani and Moosavi-Movahedi 2018). These metal ions play a key role in the enzymatic catalysis of these enzymes: while SOD isoforms use their redox metal centers at the active site to promote the dismutation of O_2_^•−^ to O_2_ and H_2_O_2_, the GPx and CATs use similar redox reactions to disproportionate H_2_O_2_ into H_2_O and O_2_ (Sarma and Mugesh 2008, Batinic-Haberle et al. 2014, Sepasi Tehrani and Moosavi-Movahedi 2018). Notably, these enzymes have been reported as potential therapeutic candidates for reducing primary oxidative events characterized by an increase in ROS and the consequent attack on biomolecules, as well as secondary events related to the redox regulation of various processes, such as inflammation (Miao and St. Clair 2009, Nandi et al. 2019).

Similar to natural enzymes, the catalytic reduction of ROS by synthetic antioxidants can be achieved using coordination compounds (Day 2009, Batinić-Haberle et al. 2010). Amongst the several advantages of using such compounds, which include reduced size, high cell permeability, increased circulating half-life, low antigenicity, and reduced manufacturing costs), the most promising properties observed in this class of compounds are their catalytic efficiency in the elimination of H_2_O_2_ and/or O_2_^−^ and their capacity to modulate cellular redox environment (Day 2009, Batinic-Haberle et al. 2014). Metalloporphyrins, macrocyclic N-containing ligands and salen derivatives are the most notorious representatives of antioxidant compounds, and promising efficacy in attenuating oxidative stress has been reported in several models of study (e.g. *in vitro* and experimental animals), including for human diseases (Tian et al. 1993, Li et al. 2003, Doctrow et al. 2012, Bonetta 2018). Due to the relevance and applicability of such a class of compounds, many other metal-based compounds have been described as synthetic antioxidants. Our group reported a series of coordination compounds synthesized with the ligand 1-[bis(pyridin-2-ylmethyl)amino]-3-chloropropan-2-ol (HPClNOL) that showed promising catalytic activity *in vitro*, and in *Saccharomyces cerevisiae* was able to reduce H_2_O_2_, O_2_^•-^ and HO^•^ toxicity and, consequently, increase cell survival under oxidative stress and ageing (Ribeiro et al. 2015, 2017, Menezes et al. 2023). Interestingly, the efficacy of these complexes appears to be related to the reduction of protein and lipid oxidation, as well as the modulation of metal homeostasis in the intracellular milieu (Ribeiro et al. 2015, 2017). More recently, a 1,10-phenantroline-octanediaoate Mn^2+^-complex was reported exhibiting significant antioxidant potential, modulating intracellular oxidation and decreasing the susceptibility of *S. cerevisiae* cells and *G. mellonella* larvae to oxidative stress (Queiroz et al. 2022). Moreover, this Mn^2+^-complex also attenuated the toxicity and aggregation of alpha-synuclein (αSyn), a human protein related to Parkinson´s disease, by interacting with negatively charged acidic residues (e.g. Asp135) located at the C-terminal region of αSyn monomers (Queiroz et al. 2022). Altogether, it is somewhat evident that synthetic antioxidants become an interesting alternative for therapeutic use against oxidative stress-related pathologies.

Herein, we investigated the potential of a series of a Fe(III), Mn(II)Mn(III) and Cu(II) coordination compounds synthesized with the N_3_O donor ligand H_2_BPClNOL, N-(2-hydroxybenzyl)-N-(2-pyridylmethyl)[(3-chloro)(2-hydroxy)]-propylamine, in protecting *Saccharomyces cerevisiae*, which was singly treated with complexes and stressed with H_2_O_2_ and menadione, an O_2_^•−^ radical generator. Additionally, we analyzed the efficacy of these compounds in extending the longevity of yeast cells using a yeast chronological aging model. This study focused on evaluating changes in cell damage as well as the activation of primary (e.g. activation of SOD and CAT) and secondary (e.g. expression and activation of heat shock protein 104–Hsp104) cellular responses after treatment with the complexes. Given its involvement in the yeast response to various stressors, including oxidative stress, we investigated whether the singular treatment of cells with the complexes could independently induce the expression of Hsp104. Finally, we explored the benefits of treatment with the complexes in the larvae of *G. mellonella*, an invertebrate model of study. The larvae were subjected to H_2_O_2_ stress, and we addressed the impact of the treatment with the complexes on the insect's life cycle.

## Materials and methods

### Synthesis of the complexes

**Figure 1.**
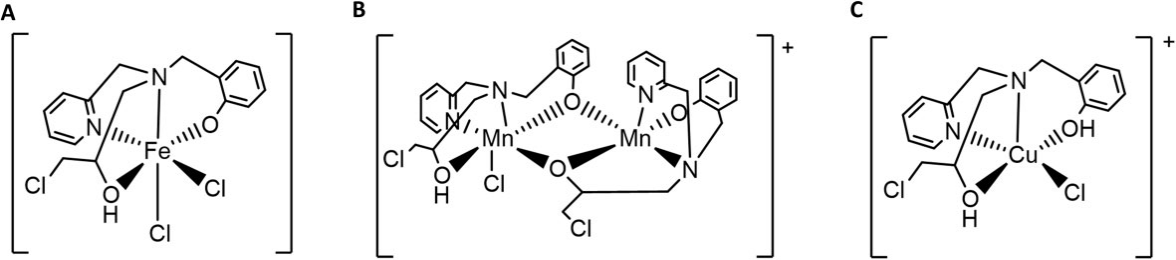
Chemical structure of the complexes 1 (A), 2 (B) and 3 (C) based on the monocrystal x-ray data.

The ligand (N-(2-hydroxybenzyl)-N-(2-pyridylmethyl) [(3-chloro)(2-hydroxy)]-propylamine) (H_2_BPClNOL) (Horn Jr. et al. 2000) and its metal-based derivatives [Fe(HBPClNOL)(Cl_2_)]·H_2_O (i) (Silva et al. 2008), Mn(µ-H_2_BPClNOL)(µ-BPClNOL)Mn(Cl)](ClO_4_)·2H_2_O (ii) (Costa et al. 2018) and [Cu(H_2_BPClNOL)Cl]Cl·H_2_O (iii) (Fernandes et al. 2010) were synthesized as previously reported (Fig. 1). Infrared and elemental (CHN) analyses were carried out and the results agree with the data reported in the literature.

### Antioxidant activity *in vitro*

For the analysis of the complex's antioxidant activity, the *in vitro* microscale DPPH radical (2,2-diphenyl-1-picrylhydrazyl) capture method was used (Mensor et al. 2001). DPPH samples initially prepared to 250 µM in ethanol were then successively diluted in the same solvent to a final concentration of 3.91, 7.82, 15.63, 31.25, 62.5, 125, and 250 µM. To avoid the interference of colors associated with the compounds, a blank system containing the complexes diluted in ethanol, at each concentration tested was also prepared. The auto reduction of DPPH was used as a control to evaluate the antioxidant percentage of the complexes. After serial dilution and controls preparation, 50 µL of DPPH was added to each well, in a 96-well microplate, except for the blank system prepared to reduce the interference related to the color of the complexes. Samples were kept protected from light at room temperature and after 30 min of reaction, the absorbance was measured at 518 nm. To calculate the RSA_50_, the *GraphPad Prism 9.0.0 software* was used. Data were normalized, and the tool “*Asymmetric Sigmoidal, 5PL, X is log(concentration)*” was applied (Chen et al. 2013).

The interaction between complexes and H_2_O_2_ or O_2_^•^−^^ was also investigated spectroscopically by UV-Vis and EPR. Stock solutions of the complexes (1 mM) were prepared in PBS (0.1% DMSO). For UV-Vis spectroscopy, 540 µL of the complex solution was added to a quartz cuvette and its spectrum was obtained. Then, 60 µL of a 1 M solution of H_2_O_2_ was added to the cuvette (ratio complex:H_2_O_2_ ∼ 1:100) and the spectra were recorded every 12 s for 5 min. For EPR studies, H_2_O_2_ (150 µL, 100 mM) was added to an EPR tube containing 150 µL of the complex to be analyzed. The ratio complex:H_2_O_2_ was1:100. Readings were carried at 120 K after 2, 30 and 60 min. To evaluate the interaction between complexes and O_2_^•−^, stock solutions (1 mM) for each complex and KO_2_ (2 and 5 mM) were prepared in DMSO. The initial spectra of each complex were recorded by UV-Vis and EPR. In UV-Vis, aliquots of 10 µL of KO_2_ (2 mM) were added to the cuvette and spectra were recorded after each addition. The reaction was also monitored through time, with the addition of a single aliquot of 100 µL of KO_2_ to the cuvette and spectra were recorded every 12 s for 5 min. For EPR studies, different volumes of KO_2_ solution were added to an EPR tube containing 150 µL of the complex stock solution. The volume was completed to 300 µL with DMSO and readings were carried out after 2 min reaction at 120 K.

### Yeast strains and growth conditions

Wild-type strain of *S. cerevisiae*, BY4741 (*MATa; his3Δ1; leu2Δ0; met15Δ0; ura3Δ0*), and its isogenic mutant *sod1Δ* (BY4741 except for *YJR104c:kanMX4*) were used to evaluate the antioxidant activity of the complexes. Stocks of these strains were maintained on solid 2% YPD (i.e. 1% yeast extract, 2% glucose, 2% peptone, and 2% agar) under appropriate conditions (Pereira et al. 2001a, 2003). For all experiments cells were grown in liquid 2% YPD (i.e. 1% yeast extract, 2% glucose and 2% peptone) to mid-exponential phase (0.8–1.0 mg dry weight/mL)—cells in fermentative metabolism—using an orbital shaker at 28°C and 160 rpm, with a 5:1 ratio of flask volume:medium (Pereira et al. 2001a, 2003).

### Oxidative stress tolerance and the adaptive treatments with 1, 2, or 3

Exponentially fermentative cells were directly exposed to oxidizing agents (1 mM H_2_O_2_ or 30 mM menadione) and incubated at 28°C and 160 rpm for 1 h (Pereira et al. 2003, Castro et al. 2007). To analyze the antioxidant activity of **1, 2**, and **3**, the cells were pretreated with increasing concentrations of the complexes for 1 h at 28°C and 160 rpm (Ribeiro et al. 2015, Queiroz et al. 2022). Next, the cells were harvested, washed twice with sterile water, resuspended in the original culture medium, previously centrifuged, and then subjected to oxidative stress. Tolerance was analyzed by plating treated and non-treated cells in 2% solid YPD medium, in triplicate, before and after oxidative stress. Control cells (non-treated and non-stressed) were used as the control for tolerance determination, calculated as the ratio between the number of colonies forming units counted after oxidative stress and the control condition.

### Detection of lipid peroxidation

Lipid peroxidation was determined by the TBARS (thiobarbituric acid reactive substances) method, which detects the final product of lipid oxidation, malondialdehyde (MDA), as previously described (Steels et al. 1994).

### Determination of catalase (CAT) and superoxide dismutase (SOD) activities

Enzyme activities were carried out in cell-free extract submitted or not to treatment with the complexes (Mariani et al. 2008). Activities were also determined in samples treated with the complexes and then exposed to oxidative stresses (H_2_O_2_ or menadione). CAT activity was performed by enzymatic kinetics following the progress of H_2_O_2_ consumption at 240 nm (Aebi 1984). Catalase activity was expressed as µmol/min/mg ptn, in which one catalase unit is defined as the amount of the enzyme that catalyzes the degradation of 1.0 µmol H_2_O_2_ per minute. Sod activity was performed by monitoring the inhibition of NBT reduction (Gamero-Sandemetrio et al. 2013). After the zymogram, the polyacrylamide gel electrophoresis was digitalized on EC3 UVP bioimaging system, and SOD activity was determined using ImageJ software, which considers the area of SOD band density. The activity was expressed as a fold increase in SOD activity, calculated by the ratio of complex treated or H_2_O_2_-stressed and control (non-treated and non-stressed) cells.

### Expression and activation of the heat shock protein, Hsp104

The expression and activation of the Hsp104 protein was monitored by fluorescence microscopy in the BY4741 *S. cerevisiae* strain genetically modified to express the GFP-tagged Hsp104 (Kroschwald et al. 2015). The expression and activation of the Hsp104 protein were investigated in cells submitted or not to a 1 h treatment with the complexes 1, 2, and 3. As a positive control, the expression and activation of the Hsp104 protein was assessed by submitting yeast cells to a mild heat-shock treatment at 40ºC/1 h. The expression of Hsp104 was determined by calculating the percentage of fluorescent cells in relation to the untreated cells. The activation of Hsp104 was determined by calculating the percentage of cells with *foci* formation, referring to Hsp104 assembling with protein aggregates.

### Chronological lifespan and complexes adaptive treatment

Chronological aging was determined in post-mitotic yeast cells, accordingly (Subramaniyan et al. 2019). Post-mitotic (3-day stationary phase) cells were chronologically aged for 28 days at 160 rpm/28ºC in the absence or in the presence of 1, 2, or 3 in the culture medium. Chronological lifespan was determined by plating aged cells every 4 days. Untreated cells plated on day 0 of the experiment (3-day stationary phase cells) were considered 100% alive.

### *Galleria mellonella* stress conditions

To analyze the susceptibility of *G. mellonella* under oxidative stress conditions, larvae from the 7^th^ instars were subjected to H_2_O_2_ (5 M) stress, which was directly injected (10 µl) in the last left pro-leg, using a Hamilton syringe, into the hemocoel of *G. mellonella* larvae (Queiroz et al. 2022). A group of *G. mellonella* larvae, previously treated with 1, 2, or 3 at different dosages (50 mg.kg^−1^, 125 mg.kg^−1^, and 250 mg.kg^−1^), was also stressed with 5 M H_2_O_2_. The treatment with **1–3** was also performed by direct injection of complexes. However, in this case, complexes were administered in the right-left pro-leg into the hemocoel of *G. mellonella* larvae 3 h before the H_2_O_2_ injection. All experiments were performed in triplicate and each group was formed by 10 larvae. As a control for mechanical injury, in all experiments, a group of 10 larvae received a direct injection with 10 µl of sterile distilled water, as described above. Survival was monitored at 24 h intervals until the end of the insect`s life cycle. Larvae that did not respond to stimulation performed by touch using a micropipette tip were considered dead.

### Statistical analysis

All experiment data were analyzed using *Graph Pad Prism 9* (San Diego, California, USA) software. All values were expressed as mean values ± standard deviation (SD) of at least three independent experiments and were analyzed using one-way Anova or two-way Anova, which denotes homogeneity between experimental groups at P ˂ 0.05.

## Results

### The *in vitro* antioxidant activity of 1, 2, and 3

**Figure 2.**
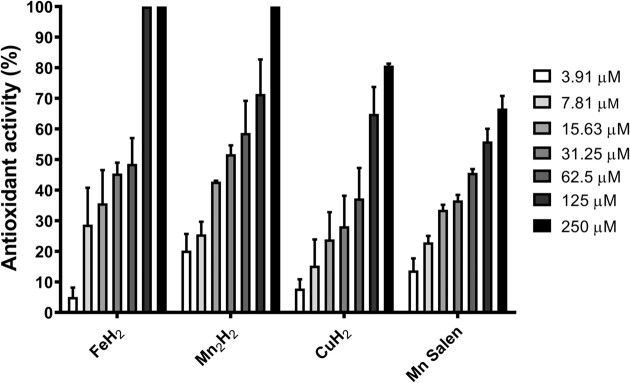
*In vitro* antioxidant activity of 1–3 complexes and [Mn(SALEN)(Cl)]. The *in vitro* evaluation of radical scavenging activity was determined by the DPPH method. Results were expressed as a percentage of the ability to reduce the DPPH radical and represent the mean ± standard deviation of at least three independent experiments.

It was reported previously that 1–3 show SOD and CAT-like activity. However, amongst these complexes, **3** showed such activity only in the presence of a co-catalyst (piperazine) (Costa et al. 2018, Guerreiro et al. 2020). Herein 1–3 were evaluated against the DPPH radical. In comparison, the well-known antioxidant complex, [Mn(SALEN)(Cl)] (EUK-8), was employed as a reference to evaluate the radical scavenging activity against DPPH. As shown in Fig. 2, the DPPH radical scavenging activity of the tested complexes increased dose-dependently. The DPPH radical was completely scavenged by the iron compound (1) at a concentration of 125 µM while the same result was reached by 2 only at 250 µM. Compound 3 decomposed around 80% of the radical at the highest concentration. Regarding the DPPH radical scavenging activity of [Mn(SALEN)(Cl)], our results showed that the profile presented by this standard antioxidant was resembled to that observed for the complex 3.

The RSA_50_ (Radical Scavenging Activity 50: concentration of the complex capable of reducing DPPH by 50%) of each complex was evaluated. As shown in Table 1, 2 presented the lower RSA_50_ (30.8 ± 0.02 µM) in comparison to 1 (64.2 ± 0.01), 3 (67.1 ± 0.03) and [Mn(SALEN)(Cl)] (64.9 ± 0.06). This result demonstrates that although 2 reached 100% antioxidant activity only at 250 µM concentration, contrasting to that observed for 1, the required concentration of 2 to reduce DPPH by 50% was lower than that presented by the other complexes. Of relevance is the fact that compound 2 exhibited superior RSA_50_ compared to [Mn(Salen)Cl], while compounds 1 and 3 demonstrated a similar RSA_50_ to the selected standard antioxidant compound.

**Table 1.** RSA_50_ values for antioxidant activity.

Complexes	EC_50_ (µM)
**1**	64.2 ± 0.01
**2**	30.8 ± 0.03*
**3**	67.1 ± 0.02
**[Mn(SALEN)(Cl)]**	64.9 ± 0.06

The calculated RSA_50_ values of complexes were obtained from a non-linear regression curve dose *versus* antioxidant activity determined by the DPPH method. RSA_50_ values of complexes were obtained as the mean ± standard deviation of at least 3 independent experiments. *Represents statistically different results in relation to **[Mn(SALEN)(Cl)]** (*P* < 0.05).

### Complexes act as antioxidants protecting *S. cerevisiae* cells from oxidative stress

To further evaluate the *in vivo* antioxidant activity of the complexes, the tolerance of the wild type strain of *S. cerevisiae*, treated or not with 1–3 was analyzed under oxidative stress generated by H_2_O_2_ (1,0 mM/1 h) and menadione (30 mM/1 h) (Fig. S1). Before assessing the antioxidant capacity of the complexes, it was verified whether *S. cerevisiae* cells would be susceptible to high concentrations of these complexes. In contrast to the chosen conditions of oxidative stress, treating cells with complexes did not impair the survival of *S. cerevisiae* cells (Fig. S2), indicating that the complexes exhibited non-toxic properties towards yeast cells.

**Figure 3.**
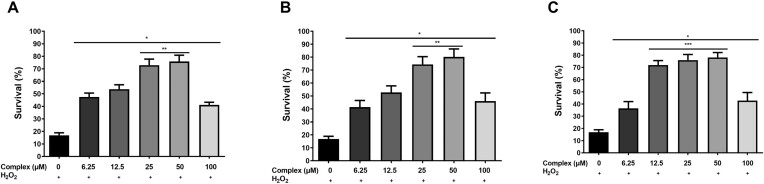
Complexes 1–3 protect *S. cerevisiae* to H_2_O_2_ stress. The wild type BY4741 cells, grown in 2% YPD medium, were treated or not for 1 h with different concentrations of **1** (A), **2** (B) or **3** (C), and then submitted to 1.0 mM H_2_O_2_ for1 h. Results are expressed as the percentage of survival and represent the mean ± standard deviation of at least three independent experiments. *Represents significantly different results compared to the positive control (1.0 mM H_2_O_2_) at *P* < 0.05. **Represents significantly different results between 25 µM and 50 µM and the other treatments with **1** or **2** at *P* < 0.05. ***Represents significantly different results between 12.5 µM, 25 µM and 50 µM and the other treatments with **3** at *P* < 0.05.

Regarding the antioxidant potential of these complexes, all compounds showed a similar pattern of cell protection. According to Fig. 3, we can observe that the treatment with the lowest concentration (6.25 µM) of the tested complexes was already able to protect yeast cells against the oxidative stress generated by H_2_O_2_. The survival of cells directly exposed to 1.0 mM of H_2_O_2_ for 1 h was 16.9%, and after previous treatments with 6.25 µM of 1–3, followed by H_2_O_2_ stress, a significant rise in the survival rates (47.5%, 41.4% and 36.4%, respectively) was observed (Fig. 3A–C). The most significant cellular protection against exposure to H_2_O_2_ was achieved under treatment employing 25 µM and 50 µM of the complexes. However, compound 3, at a concentration of 12.5 µM, could also confer cell protection at the same rates observed for the treatments with 25 µM and 50 µM (Fig. 3C). Unexpectedly, treating cells with 100 µM of all tested complexes decreased cell protection compared to the group of cells treated with 25 and 50 µM. However, the survival of cells treated with 100 µM was still higher than those directly stressed with H_2_O_2_ (1 mM).

**Figure 4.**
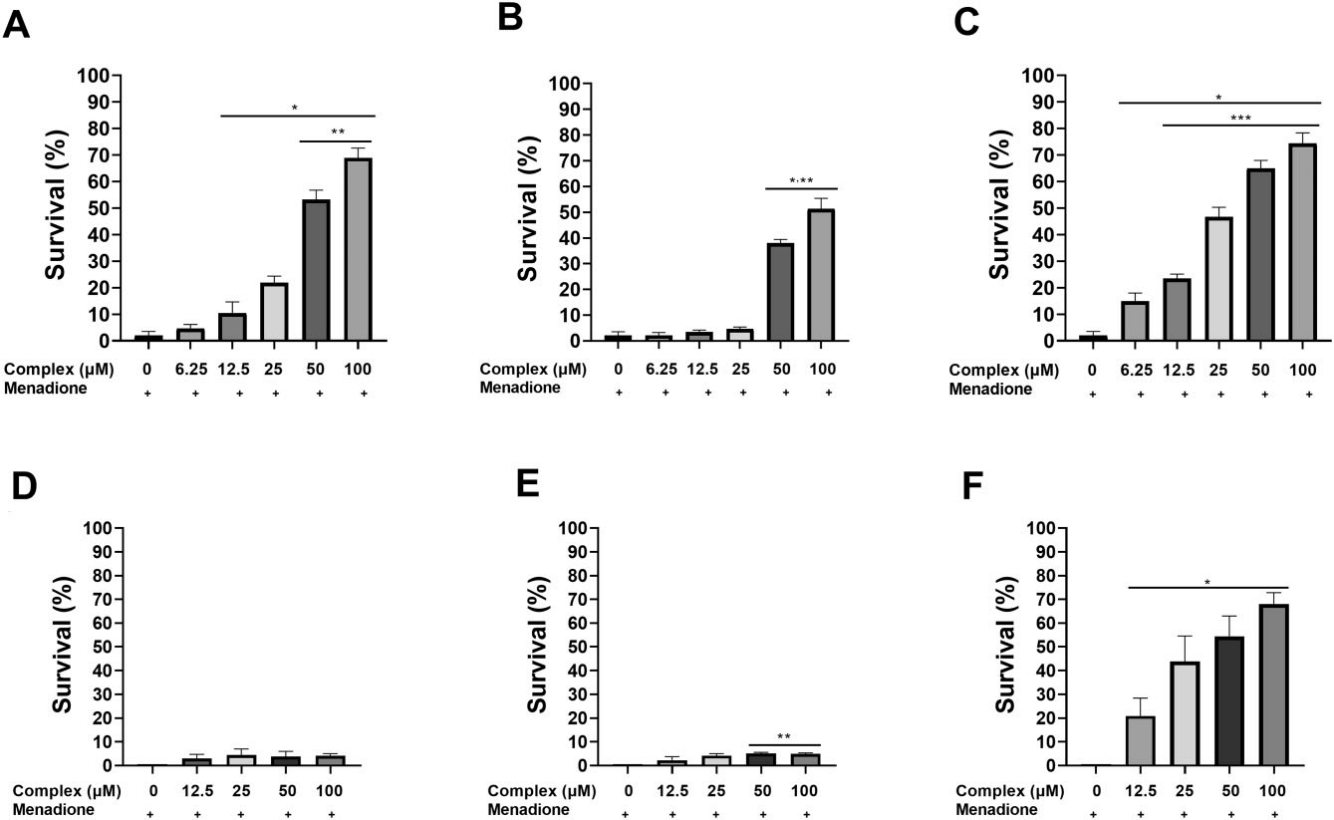
Complexes 1–3 protect *S. cerevisiae* to O_2_^•−^ stress. Cells of the wild type BY4741 strain, treated or not for 1 h with different concentrations of 1 (A), 2 (B) or 3 (C), were subjected to 30 mM menadione/1 h. Cells lacking the Sod1 enzyme (*sod1Δ*), treated or not for 1 h with different concentrations of 1 (D), 2 (E) or 3 (F), were subjected to 30 mM menadione for 1 h. Results are expressed as the percentage of survival and represent the mean ± standard deviation of at least three independent experiments. *Represents significantly different results compared to the positive control (30 mM Menadione) at *P* < 0.05. **Represents significantly different results between 50 µM and 100 µM and the other treatments with 1 or 2 at *P* < 0.05. ***Represents significantly different results between 12.5 µM, 25 µM, 50 µM and 100 µM and the other treatments with **3** at *P* < 0.05.

Since all the complexes protected yeast cells against H_2_O_2_ stress, we further investigated whether these complexes would also be able to protect cells of the wild type and its isogenic mutant deficient in the synthesis of the Cu,Zn-Sod (*sod1Δ*) from the stress generated by menadione (30 mM/1 h), an O_2_^•−^ generating drug. After menadione stress, cells of the wild type strain were drastically affected, presenting only 2.0% survival under O_2_^•−^ stress (Fig. 4A-C). The treatment with 1 or 3 increased the survival of the wild type strain in a dose-dependent manner (Fig. 4A and C). On the other hand, the treatment with 2 presented a distinct profile and did not protect the wild type cells up to the treatment with 25 µM; however, after treatment with 50 µM and 100 µM, we observed a significant increase in yeast protection when compared to the untreated cells (Fig. 4B). As expected, the lack of Cu,Zn-Sod in *sod1Δ* cells conferred high susceptibility to O_2_^•−^ stress (Fig. 4D-F). Contrasting to what was observed in the wild type strain, *sod1Δ* cells acquired tolerance to O_2_^•−^ stress only after pretreatment with complex **3** (Fig. 4F), while 1 and 2 were ineffective in protecting these mutant cells under O_2_^•−^ stress (Fig. 4D and E).

### Complexes treatment reduces lipid oxidation and differentially modulates endogenous SOD and CAT antioxidant activities

**Figure 5.**
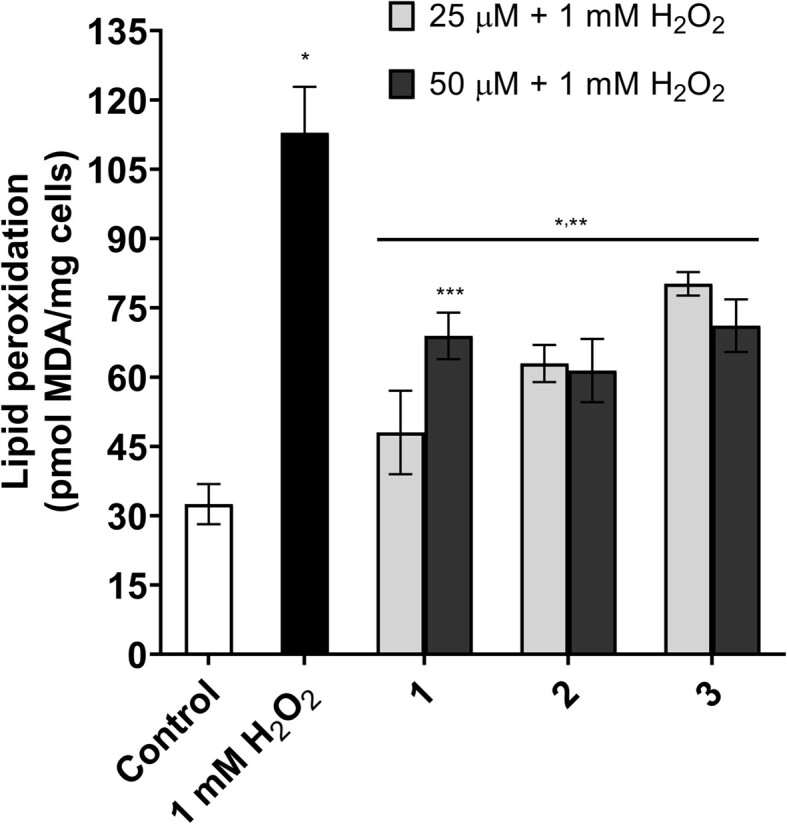
Reduction of lipid peroxidation promoted by treatments with complexes 1–3. Cells of the wild type BY4741 strain, treated or not for 1 h with different concentrations of **1, 2** or **3**, were subjected to H_2_O_2_ stress, and then prepared for the determination of lipid peroxidation. Results are expressed as pmol of MDA/mg of cell and represent the mean ± standard deviation of at least three independent experiments. *Represents significantly different results in relation to the control (*P* < 0.05). **Represents significantly different results between the treatments and the positive control (1.0 mM H_2_O_2_) at *P* < 0.05. ***Represents significantly different results between 50 µM and 25 µM treatments at *P* < 0.05.

The increase in cell survival after oxidative stress may result from reducing oxidative damage and/or eliminating ROS by treatment with 1–3. Then, we next investigated whether treatment with the complexes would be able to attenuate the lipid peroxidation promoted by stress with H_2_O_2_. As shown in Fig. 5, it is possible to observe that oxidative stress caused a drastic increase in lipid oxidation (i.e. a 3.4-fold increase) compared to the non-stressful condition. After treatment with the complexes, a significant decrease in the levels of lipid oxidation could be observed; however, this reduction was not able to restore the lipid oxidation levels observed in unstressed cells (Fig. 5). Moreover, we did not observe any differences regarding the capacity of complexes to reduce yeast lipid oxidation under H_2_O_2_ stress. All complexes were able to reduce lipid oxidation to the same magnitude.

**Figure 6.**
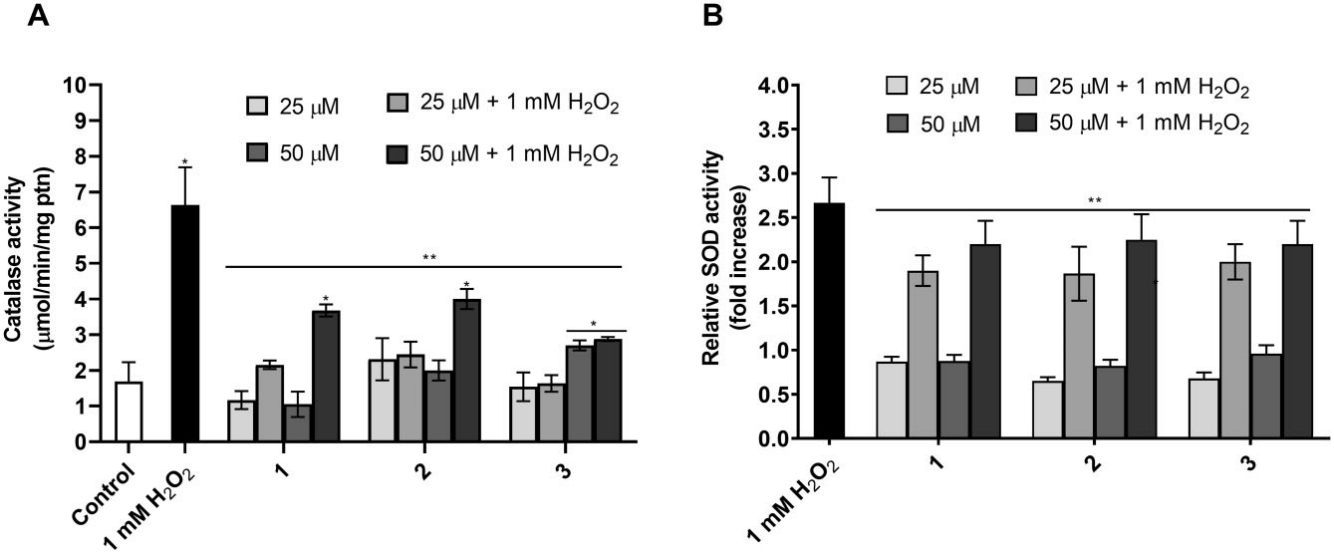
Complexes 1–3 mitigate the requirement for CAT and SOD activity under oxidative stress. For catalase activity, cell extracts were prepared for enzymatic determination immediately before and after treatments with H_2_O_2_ (A). Results were expressed as specific activity (µmol/min/mg ptn). For SOD activity, exponentially growing cells were subjected to treatment (B), and/or stress with 1 mM of H_2_O_2_. Results were expressed as a fold increase calculated by the ratio of SOD activity between cells treated with different complexes and the control condition. All results represent the mean ± standard deviation of at least three independent experiments. *Represents significantly different results in relation to the control (*P* < 0.05). **Represents significantly different results between the treatments and the positive control (1.0 mM H_2_O_2_) at *P* < 0.05.

Next, the activities of CAT and SOD were investigated to assess whether complex treatment could modulate the activities of these ROS-scavenging enzymes. As expected, cells undergoing fermentative metabolism showed low levels of CAT activity. In contrast, after submitting the cells to oxidative stress (1 mM H_2_O_2_/1 h), an increase (i.e. 3.2 times) in CAT activity was observed (Fig. 6A and B). Treating the cells with the complexes did not increase CAT activity at the tested concentrations (25 µM and 50 µM) (Fig. 6A). Interestingly, after treatments with the complexes and subsequent exposure to H_2_O_2_ stress, we observed that CAT activity presented a similar trend that was found for the unstressed cells (control) (Fig. 6A). This result might suggest that the 1–3 might deal with the stressor agent (H_2_O_2_), avoiding the requirement of CAT activation.

Regarding SOD activity, we observed a 2.7-fold increase in the activity of this antioxidant enzyme after H_2_O_2_ stress when compared to unstressed cells (Fig. 6B). Unlikely to CAT profile, the SOD activity did not change after singly treatments with the tested complexes, and the SOD activity, after 1–3 treatments followed by H_2_O_2_ stress remained as high as observed in H_2_O_2_^–^ stressed cells (Fig. 6B)

### Singly treatments with 1-3 induced Hsp104 expression, albeit no changes in its activity were observed

**Figure 7.**
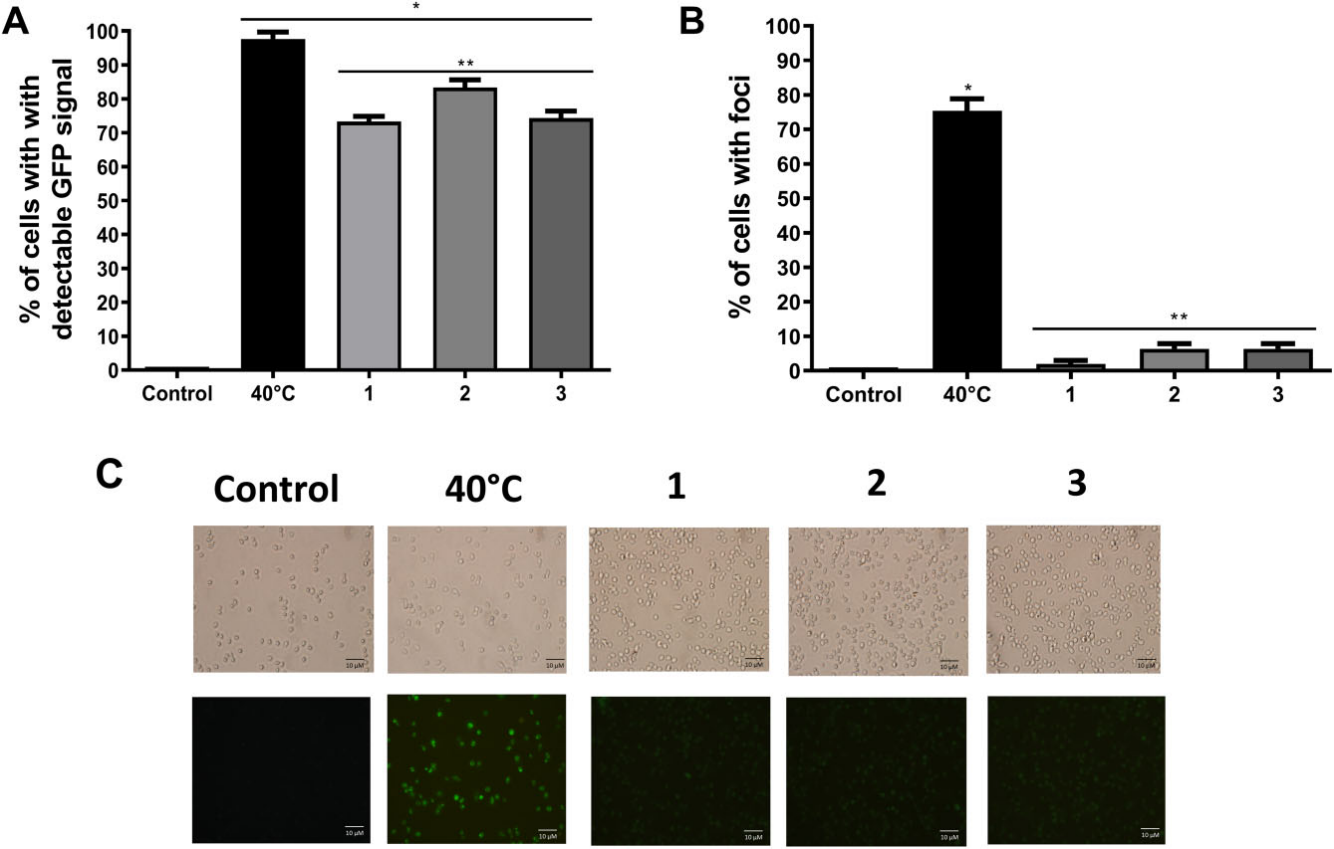
Induction and activation of Hsp104-GFP expression. Cells were treated with 50 µM of the compounds for 1 h or subjected to heat stress (40°C for 1 h), and then fluorescence was observed under a fluorescence microscope. (A) The percentage of cells expressing Hsp104. (B) The percentage of cells with detectable *foci*. (C) Representative fluorescent images of Hsp104-GFP obtained by fluorescent microscopy. Results were expressed as % of cells with Hsp104-GFP expression (A) and as % of cells with Hsp104-GFP *foci*, representing the mean ± standard deviation of at least three independent experiments. *Represents significantly different results in relation to the control (*P* < 0.05). **Represents significantly different results between the mild heat treatment (40°C/1 h) and the treatment with 50 µM of the compounds at *P* < 0.05.

To determine the expression of the Hsp104 protein after treatment with the tested complexes, the BY4741 strain was genetically modified to express the Hsp104 fused to the green fluorescent protein, GFP (Hsp104-GFP). This construction allowed us to analyze the Hsp104 expression (i.e. increase in fluorescence) and activation (i.e. increase in *foci* formation) after singly treatments with the complexes. According to Fig. 7, we can see that under normal growth conditions, no fluorescence signal emitted by the Hsp104-GFP construct was observed. This result shows that under our experimental condition the Hsp104 expression is not inducible and is not required under non-stressful conditions (Fig. 7A and C). As expected, the mild heat stress (40 ºC/1 h) induced an increase in both the expression and the activation of Hsp104 (Fig. 7A, B and C). Moreover, foci formation was distinctly observed in 75% of fluorescent cells. Interestingly, the singly complex treatments increased GFP fluorescence, indicating that these complexes promoted the induction of Hsp104 expression in yeast cells. However, it was observed that the complexes did not activate Hsp104, as evidenced by the absence of foci formation in fluorescence microscopy.

### Complexes treatment extends yeast life span

**Figure 8.**
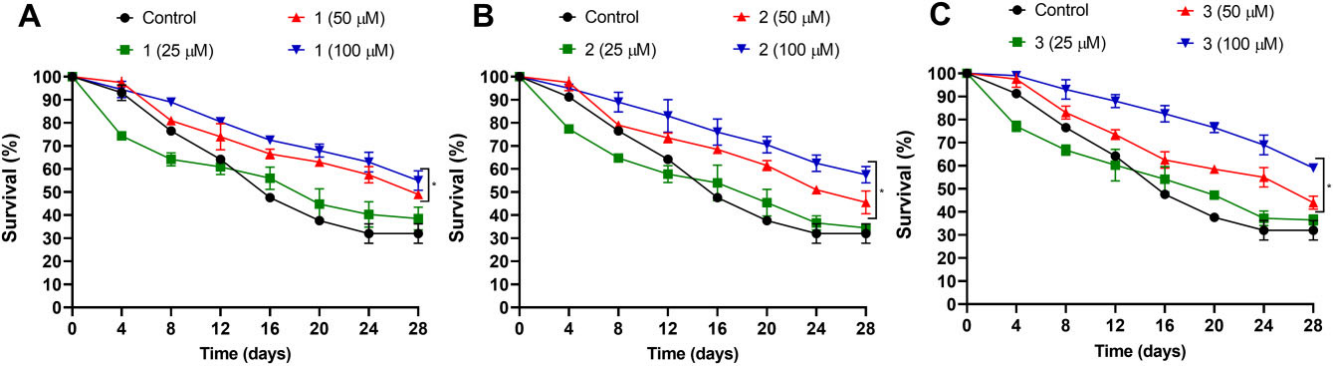
Chronological aging lifespan extension of BY4741 cells after treatment with the complexes. Cells of the wild type BY4741 strain, treated or not with different concentrations of **1** (A), **2** (B) and **3** (C) complexes, were submitted to chronological aging for 28 days. Aliquots were removed every 4 days, and the cells were plated with colonies counted after 72 h. Results are expressed as a percentage of survival and represent the mean ± standard deviation of at least three independent experiments. (*P* < 0.05). *Represents significantly different results in relation to the control (*P* < 0.05).

Since all complexes increased yeast tolerance under oxidative stress conditions, we further investigated whether the tested complexes could increase yeast lifespan during chronological aging. Lifespan was assessed in chronologically aged cells of the wild type strain of *S. cerevisiae*, BY4741, which was treated or not with the complexes (25, 50 and 100 µM). Chronological aging was monitored for 28 days, and every 4 days, the cells were collected and plated in 2% YPD medium to assess cell viability. We can see those treatments with the complexes at the highest concentrations (50 or 100 µM) extended yeast lifespan during chronological aging (Fig. 8A–C). After 28 days of chronological aging, the non-treated aged cells showed a cellular lifespan of 32%, whilst the lifespan of cells that were treated with 50 µM or 100 µM of each complex significantly increased to 44%–49% and 55%–59%, respectively (Fig. 8A–C). Moreover, our results showed that the antiaging profile of these complexes was dose-dependent since the treatment of cells with the lower concentration of complexes was somewhat similar to untreated aged cells.

### Complexes decrease *G. mellonella* larvae susceptibility to H_2_O_2_ stress and did not alter the insect life cycle

**Figure 9.**
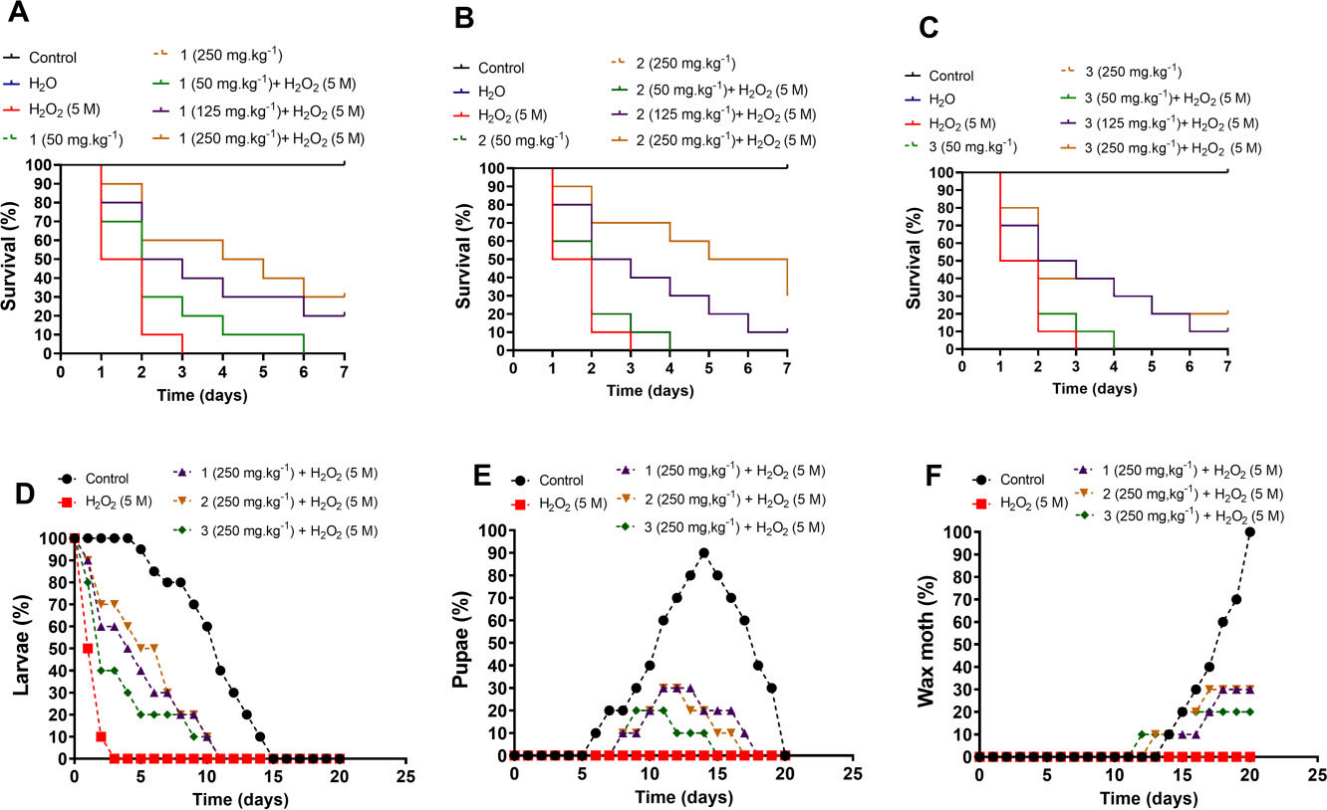
Complexes treatment reduce *G. mellonella* larvae susceptibility to H_2_O_2_ stress without interfering in larvae life cycle. *Galleria mellonella* larvae exposed to treatment with 1 (A), 2 (B) and 3 (C) complexes were subjected to stress with 5 M H_2_O_2_ for 1 h. The graphs track larvae survival (9A-C) over a 7-day period and depict the overall development throughout the entire insect life cycle (9D-F). We can observe the three stages: larvae (D), pupae (E), and moth (F). The graphs track the continuity of the animal's life cycle over 21 days. Results represent the mean ± standard deviation of at least three independent experiments.

Finally, the antioxidant potential of the complexes was investigated in the *Galleria mellonella* model of study, which was subjected to acute oxidative stress by direct injection of H_2_O_2_ into the larval hemocoel. As shown in Fig. 9 (A, B and C), all H_2_O_2_-stressed larvae died up to the 3^rd^ day of injection. Susceptibility to H_2_O_2_ was partially reverted after treatment with the complexes 1, 2 or 3 (Fig. 9 A, B, and C). The group of *G. mellonella* larvae treated with 50 mg kg^−1^ of complexes showed remarkable survival to H_2_O_2_ stress than untreated larvae. Survival increased at 1 and 3 days after treatments with complexes 2 or 3 and 1, respectively (Fig. 9 A, B, and C). By increasing the dose to 125 mg kg^−1^, we observed that the survival of *G. mellonella* larvae was improved, reaching 20% survival for 1 and 10% for 2 or 3 on the 7^th^ day after H_2_O_2_ injection. Finally, treatment of larvae with 250 mg kg^−1^ of 1 or 2 increased larvae survival to 30% on the 7^th^ day of monitoring, whilst the same treatment with **3** increased larvae survival to 20%. Therefore, our results show that the percentage of *G. mellonella* larvae that survived H_2_O_2_ stress increased considerably with previous treatment with the antioxidant complexes. As expected, the single administration of 1–3 did not affect larvae survival, having no impact on the insect's life cycle (Fig. S7).

The differentiation process from larva to moth that occurred during the insect's life cycle was also monitored after administration of H_2_O_2_, with and without treatment with the complexes. The demonstration that complexes could not interfere with the *G. mellonella* life cycle was also crucial to confirm that the treatment with these complexes was safe for the insect. As shown in Fig. 9, all the larvae of the control group reached the last stage of the insect´s life cycle, and after 20 days of follow-up, the larvae that had turned into pupa, between 6th and 14th days, differentiated into a moth. Since all larvae exposed to oxidative stress died on the third day of exposure, it was impossible to assess the interference of H_2_O_2_ in the *Galleria mellonella* life cycle. In contrast, all larvae treated with the highest dose of complexes (250 mg.kg^−1^) and survived until the 7 days of H_2_O_2_ stress were able to differentiate into a pupa between the 8th and 11th day of life. From the 13th day of life, the pupa gives rise to the adult insect, the moth (Fig. 9D–F).

## Discussion

The antioxidant potential of natural or synthetic substances is characterized by their effective ability to eliminate or, at least, attenuate the reactivity of ROS. However, substances capable of modulating the activity of endogenous protective factors and/or reducing oxidative damage can also be considered antioxidants of relevance. Herein, examining the antioxidant potential of three complexes containing iron (1), manganese (2) and copper (3) synthesized with the ligand N-(2-hydroxybenzyl)-N-(2-pyridylmethyl)[(3-chloro)(2-hydroxy)]-propylamine (H_2_BPClNOL), we showed that all complexes protected *S. cerevisiae* and *G. mellonella* from oxidative stress.

Further than the SOD and CAT-like activities previously reported for the compounds (Figs S3–S6) (Costa et al. 2018), we investigated whether the complexes would have some discernible antioxidant activity on the DPPH radical. A significant radical scavenging activity (RSA) was observed after incubating DPPH with all tested complexes. The EC_50_ value revealed that (2) presented the lowest EC_50_ compared to the other complexes, which is almost 50% lower than the result presented by 1 and 3. Considering that compound 2 has two metal ions (Mn) and also two molecules of the ligand while the others have just one, the best activity displayed by 2 may be due to its higher metal and ligand amount. Therefore, considering the concentration of metal and ligand molecules, the compounds showed similar RSA activities on DPPH.

Moreover, employing UV-Vis and EPR spectroscopy, we could understand the interaction of the complexes 1 and 3 with the H_2_O_2_ and O_2_^−•^, the stressor agents used in this study. Such investigation has already been reported for compound 2 (Costa et al. 2018). Spectroscopically, compounds 1 and 3 show different behavior in the presence of H_2_O_2_. Both UV-Vis and EPR indicate that compound 1 undergoes reduction in the presence of H_2_O_2_ since the LMCT phenolateáFe(III) disappeared and the EPR signal associated with the Fe(III) center decreased significantly (Figs S3 and S4). In controversy, the spectral features of the copper compounds did not change in the presence of H_2_O_2_.

Concerning the interaction of **1** with O_2_^−•^ the UV-Vis indicates possible coordination of the superoxide to the iron compound. The EPR also supports this interpretation since at a ratio of 1:1 (**1**:O_2_^−•^), the signal associated with Fe(III) becomes more symmetric and at a ratio of 1:2 it disappears, which indicates the reduction of the Fe(III) to Fe(II) (Figs S4 and S5). The EPR data related to the interaction of **3** with O_2_^−•^also support that a redox process is taking place since the signal associated with the Cu(II) decreased with the increase in the O_2_^−•^ concentration, indicating the formation of Cu(I). Furthermore, a new pattern at g∼2.0 has emerged at a ratio **3**:superoxide 1:4, which is attributed to the intermediated Cu(I)- O_2_^•−^ (Fig. S5) (Menezes et al. 2023). UV-Vis spectroscopy showed a new band at 450 nm, attributed to a LMCT O_2_^•−^áCu(II) (Fig. S4) (Fujisawa et al. 1994, Abe et al. 2019, Quek et al. 2021, Menezes et al. 2023).

We have previously reported the antioxidant potential of coordination compounds containing the same metal ions (Fe, Mn and Cu) but synthesized with different ligand 1-[bis(pyridin-2-ylmethyl) amino]-3-chloropropan-2-ol (HPClNOL) using the yeast *S. cerevisiae* model of study (Ribeiro et al. 2015, 2017, Thornton et al. 2016). We described that besides having *in vitro* antioxidant capacity, all the complexes investigated also protected *Saccharomyces cerevisiae* from acute H_2_O_2_ and O_2_^•−^ stresses. In our previous studies (Ribeiro et al. 2015), the antioxidant protection follows the order Fe > Cu > Mn complexes. In contrast, in this work, the copper complex showed the best protection. Of relevance is the fact that **3** efficiently protected the *sod1Δ* mutant strain, which lacks the Cu,Zn-SOD (SOD1) antioxidant enzyme, when exposed to oxidative stress. This result is very interesting since **3** seems to functionally replace the antioxidant enzyme Cu,Zn-SOD, rescuing the high susceptibility phenotype of this mutant strain. Moreover, this protective profile was somewhat similar to that obtained for the complexes containing the HPClNOL ligand series. In this context, it is worth mentioning that several synthetic antioxidants, including [Mn(Salen)Cl], failed to revert sod1 deficiency [38], and amongst all synthetic antioxidants tested (MnSalen, Mn-macrocyclic Mn-porphyrin derivatives) only MnTM-2-PyP was effective to rescue *sod1Δ* defects [38].

High levels of ROS are intimately related to the accumulation of oxidative damage, which leads to the onset of aging and age-related diseases (e.g. neurodegenerative diseases and cancer) (Cadenas and Davies 2000, Tan et al. 2018). Therefore, there has been a growing interest in antioxidant compounds to attenuate the effect of oxidative stress and aging. In our study, we observed that all compounds extended yeast lifespan, even though no statistical differences were observed between these complexes. Similar antiaging results were obtained by testing other synthetic antioxidants (Ribeiro et al. 2015, 2017). In this context, although we did not observe any differences between the complexes reported here, our results seem promising since the treatment of *C. elegans* with the synthetic antioxidants EUK-8 and EUK-134, capable of increasing the endogenous SOD activity did not extend the nematode lifespan (Keaney et al. 2004).

Examining the activity of SOD and CAT antioxidant enzymes, the expression of HSP104 and the lipid peroxidation profile, we observed that the compounds might also be involved in cellular reprogramming to maintain intracellular redox status. Activation of antioxidant enzymes is a common mechanism for the acquisition of resistance to oxidative stress. In *S. cerevisiae*, it has been reported that *de novo* synthesis of antioxidant enzymes increases under ROS *stimuli* (Moye-Rowley 2002, Herrero et al. 2008). Amongst the transcription factors involved in the response of *S. cerevisiae* to stress conditions, the transcription factors Yap1 and Hsf1 are the major sensors and signal transducers that regulate gene expression in response to oxidative stress (Morano et al. 2012, Mejía-Barajas et al. 2017, Rodrigues-Pousada et al. 2019). Thus, activating antioxidant enzymes and/or heat shock proteins (HSP´s) is critical for the acquisition of resistance against oxidative stress (Pereira et al. 2001b, 2003). Contrasting with the results reported in the literature for [Mn(Salen)Cl] (Keaney et al. 2004), we did not detect any increase in SOD and/or CAT activity after the treatment with the complexes. Interestingly, catalase activity was reduced after treating yeast cells with the complexes and then subjected to oxidative stress. This result strongly points to the antioxidant activity of the complexes that, by maintaining the redox balance at low levels, no longer requires CAT activity to be functional in response to oxidative stress. Several studies reported that HSP`s plays crucial roles in oxidative stress response, perhaps due to their modulatory effects on inflammation cascades that control ROS generation and/or the chaperone activities assisting misfolded protein to refold properly (Vacher et al. 2005, Hauet-Broere et al. 2006, Grimminger-Marquardt and Lashuel 2010, Morano et al. 2012). In this work, the expression of Hsp104 was monitored by the increase in fluorescent cells after treatments, while activation was determined by the formation of foci, characterized by the misfolding and aggregation of proteins. Indeed, the induction of Hsp104 expression was highly detectable in cells treated with mild heat-shock (40°C/1 h), and to a lesser extent in cells treated with the complexes. However, foci formation was observed only in heat-treated cells. The absence of foci formation in cells treated with the complexes was expected since the exposure of cells to the complexes was non-toxic and, therefore, incapable of inducing protein misfolding and aggregation. These results suggest that the complexes may be triggering a specific cellular signaling pathway that leads to the *de novo* synthesis of HSP's, essential for adaptation and additional protection against a subsequent stress condition.

Lipid peroxidation caused by oxidative stress conditions, as well as the reduction of this process by antioxidant substances, has been studied by our group (Herdeiro et al. 2006, Horn et al. 2010, Ribeiro et al. 2015). The use of antioxidant complexes as an alternative to reduce lipid peroxidation has already been addressed in a previous study, whose results showed that treating *S. cerevisiae* with complexes of the HPClNOL series reduced lipid peroxidation values by more than 50% (Ribeiro et al. 2015). Contrasting with reported data, in which we showed that the Fe(III) compound was slightly more efficient in reducing lipid peroxidation, herein, no significant changes were observed between the tested complexes. Other antioxidant complexes have also shown a positive effect in reducing lipid peroxidation in different models of study (Gonzalez et al. 1995, Zhang et al. 2004, Clausen et al. 2010). The MnSalen derivatives EUK-189 and EUK-207 reduced lipid peroxidation by almost 50% in mouse brains subjected to aging for 11 months (Liu et al. 2003). Another study showed that treatment with MnSalen derivatives compounds decreased lipid peroxidation levels in mice fed a diet that caused nonalcoholic steatohepatitis (Rezazadeh et al. 2012).

The use of *G. mellonella* has been rapidly disseminated through different areas of science (Champion et al. 2016, Fernandes et al. 2017). The similarity between the innate immune system of *G. mellonella* larvae and humans puts this invertebrate in focus as an alternative model for studies on the effect of drugs and fungal/bacterial infection (Kellett et al. 2011, Adamski et al. 2014, Trevijano-Contador and Zaragoza 2018). Using *Galleria mellonella* larvae we proved that the complexes act as antioxidants and are safe in terms of their toxicity. We have recently reported the use of *G. mellonella* larvae to investigate the antioxidant potential of a Mn^2+^-complex, [Mn_2_(μ_2_-oda)(phen)_4_(H_2_O)_2_]^2+^, which reduced the susceptibility of this invertebrate model to H_2_O_2_ stress (Queiroz et al. 2022). Unquestionably, regardless of the treatment dose used, all complexes tested could prolong the survival of *G. mellonella* larvae. In addition, the complexes also ensured the continuity of the insect's life cycle: all larvae that survived the H_2_O_2_ stress reached the adult stage of the life cycle, the moth. This is the first time that monitoring *G. mellonella* life cycle (i.e. development from larvae to pupae and then to moth) has been employed to assess the toxicity, safety of use, and antioxidant activity of coordination compounds.

## Conclusions

In this study, we carried out *in vitro* and *in vivo* measurements to assess the antioxidant potential of three complexes synthesized with the ligand H_2_BPClNOL. Our results revealed that these complexes showed suitable antioxidant activities by reducing the susceptibility of *S. cerevisiae* and *G. mellonella* to oxidative stress. In this regard, the increased survival observed in *S. cerevisiae* treated with the synthetic antioxidants seems to be strongly related to the maintenance of redox homeostasis (e.g. reduction of lipid oxidation and modulation of SOD and CAT activity) and activation of cell stress response (e.g. induction of HSP104 expression). We also observed that *S. cerevisiae* and *G. mellonella* well-tolerated these complexes, which in our experimental conditions were non-toxic for both models of study. Inclusive, all *G. mellonella* larvae that were treated with the complexes and survived oxidative stress could complete their life cycle, differentiating from larva to pupa and then from pupa to moth. Our results suggest that the complexes may be also triggering a specific cell signaling pathway that culminates in *de novo* synthesis of protective factors such as HSP's required for further protection against a sudden stress condition. Taken together, our results point to a promising antioxidant potential of studied complexes to circumvent the harmful of oxidative stress.

This study showed a new exciting aspect related to the antioxidant activity of coordination compounds by modulating the cellular response, which raises interest in whether treatment with these compounds would be able to attenuate, in alternative models of study, the cellular dysfunctions observed in human pathologies. We hope to address such questions in future work.

## Credit authorship contribution statement

*Larissa M. M Mattos :* Investigation, Methodology, Formal analysis, Visualization, Writing—original draft, Writing—review & editing. *Hyan M. Hottum :* Investigation, Formal analysis. *Daniele C. Pires :* Investigation, Formal analysis. *Bruna B Segat :* Synthesis and characterization of the complexes, EPR measurements and review. *Adolfo Horn Júnior :* Investigation, Formal analysis, Writing—review & editing, Resources. *Christiane Fernandes Horn:*Formal analysis, Investigation, Writing—review & editing, Resources. *Marcos Dias Pereira:* Conceptualization, Project administration, Supervision, Resources, Funding acquisition, Visualization, Writing—review & editing.

## Supplementary Material

foad052_Supplemental_File

## Data Availability

The data generated and analyzed during this study are available from the corresponding author on reasonable request.
