# Supplementary material for: Exploring the antioxidant activity of Fe(III), Mn(III)Mn(II), and Cu(II) compounds in Saccharomyces cerevisiae and Galleria mellonella models of study
Source: FEMS Yeast Res. 2023 Dec 20;24:foad052. doi: 10.1093/femsyr/foad052 (PMC10776354; doi:10.1093/femsyr/foad052)
Supplement: foad052_Supplemental_File [file foad052_supplemental_file.docx]

**Suplementary material**


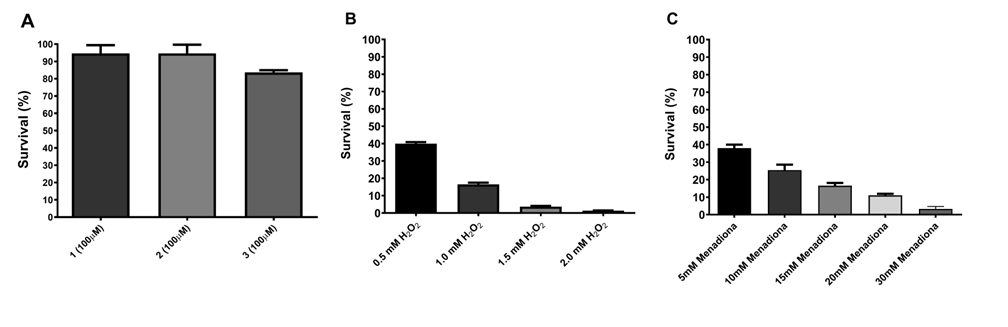


**Figure S1. Toxicity of 1-3 complexes, H_2_O_2_ and menadione in *S. cerevisiae*.** Cells of the wild type BY4741 strain grown in 2% YPD medium were used to evaluate the complex cytotoxicity in the presence and absence of the complexes. (A) Survival of the BY4741 strain after treatment with 100 μM of the complexes for 1 h. (B) Survival of the BY4741 strain after oxidative stress caused by different concentrations of H_2_O_2_ for 1 h. (C) Survival of the BY4741 strain after oxidative stress caused by different concentrations of menadione for 1 h. Results are expressed as percent survival and represent the mean of three independent experiments.


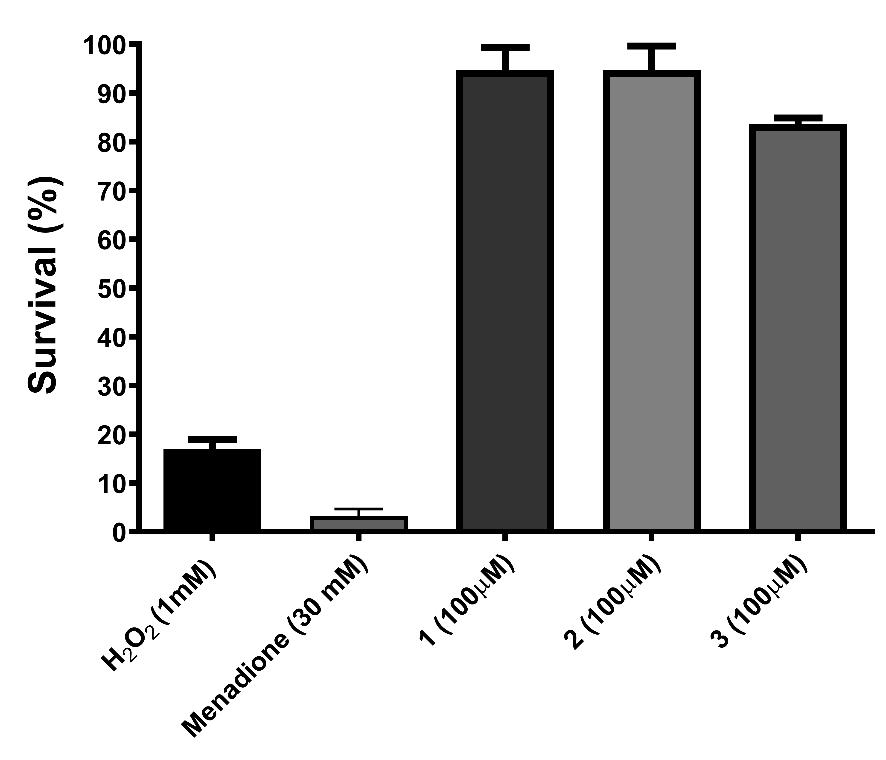


**Figure S2. Toxicity of H_2_O_2_ and menadione compared to 1-3 complexes in *S. cerevisiae*.** Cells of the wild type BY4741 strain grown in 2% YPD medium were used to evaluate the toxicity of H_2_O_2_ (1 mM), menadione (30 mM) and 1-3 complexes. Results are expressed as percent survival and represent the mean of three independent experiments.

 **Figure S3. UV-Vis spectra of complex 1 (left) and 3 (right) with H_2_O_2_ after different times of reaction.**

**Figure S4. EPR spectra of complex 1 (left) and 3 (right) with H_2_O_2_ after different times of reaction.**

 **Figure S5. UV-Vis spectra of complex 1 (left) and 3 (right) with O_2_^-.^ after different times of reaction.**

 **Figure S6. EPR spectra of complex 1 (left) and 3 (right) with different metal:O_2_^-.^ ratios, as indicated.**


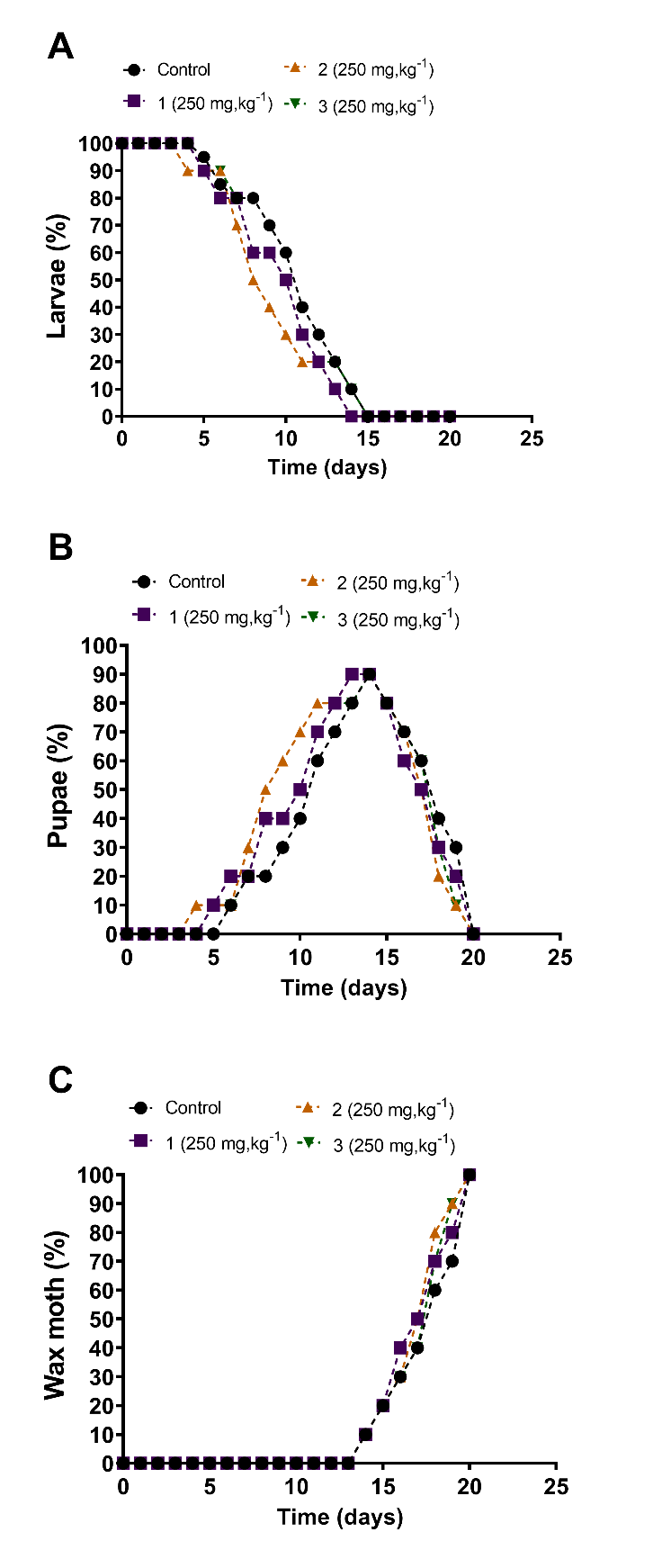


**Figure S7. Treatment with the complexes did not affect the life cycle of *Galleria mellonella*.** *Galleria mellonella* larvae were exposed to treatment with complexes **1,** **2** and **3** and the life cycle was monitored. The graphs track the continuity of the insect's life cycle over 21 days.
